# Forecasting Implementation, Adoption, and Evaluation Challenges for an Electronic Game–Based Antimicrobial Stewardship Intervention: Co-Design Workshop With Multidisciplinary Stakeholders

**DOI:** 10.2196/13365

**Published:** 2019-06-04

**Authors:** Enrique Castro-Sánchez, Anuj Sood, Timothy Miles Rawson, Jamie Firth, Alison Helen Holmes

**Affiliations:** 1 National Institute for Health Research Health Protection Research Unit in Healthcare-Associated Infection and Antimicrobial Resistance Imperial College London London United Kingdom; 2 Jamie Firth Consultancy Ltd London United Kingdom

**Keywords:** serious games, antimicrobial stewardship, medical education

## Abstract

**Background:**

Serious games have been proposed to address the lack of engagement and sustainability traditionally affecting interventions aiming to improve optimal antibiotic use among hospital prescribers.

**Objective:**

The goal of the research was to forecast gaps in implementation, adoption and evaluation of game-based interventions, and co-design solutions with antimicrobial clinicians and digital and behavioral researchers.

**Methods:**

A co-development workshop with clinicians and academics in serious games, antimicrobials, and behavioral sciences was organized to open the International Summit on Serious Health Games in London, United Kingdom, in March 2018. The workshop was announced on social media and online platforms. Attendees were asked to work in small groups provided with a laptop/tablet and the latest version of the game On call: Antibiotics. A workshop leader guided open group discussions around implementation, adoption, and evaluation threats and potential solutions. Workshop summary notes were collated by an observer.

**Results:**

There were 29 participants attending the workshop. Anticipated challenges to resolve reflected implementation threats such as an inadequate organizational arrangement to scale and sustain the use of the game, requiring sufficient technical and educational support and a streamlined feedback mechanism that made best use of data arriving from the game. Adoption threats included collective perceptions that a game would be a ludic rather than professional tool and demanding efforts to integrate all available educational solutions so none are seen as inferior. Evaluation threats included the need to combine game metrics with organizational indicators such as antibiotic use, which may be difficult to enable.

**Conclusions:**

As with other technology-based interventions, deploying game-based solutions requires careful planning on how to engage and support clinicians in their use and how best to integrate the game and game outputs onto existing workflows. The ludic characteristics of the game may foster perceptions of unprofessionalism among gamers, which would need buffering from the organization.

## Introduction

### The Threat of Drug-Resistant Infections

Although antimicrobial resistance is an evolutive phenomenon that cannot be stopped [1], reducing inadequate use of antibiotics would prolong their effectiveness and mitigate the clinical, human, and economic costs of drug-resistant infections [2]. As an example of such costs, in 2015 an estimated 672,000 infections with antibiotic-resistant bacteria were reported in the European Union [3], and yearly drug-resistant infection–attributable mortality worldwide has been forecasted to reach 10 million people by 2050 [4,5].

Antimicrobial stewardship (AMS) fosters the optimal use of antibiotics by health care professionals, patients, and citizens [6], combining organizational, structural, behavioral, and educational components. Several initiatives have reported on educational resources and interventions focused on undergraduate human health and veterinary students [7] as well as existing health care workers [8]. These interventions aimed to address existing gaps in undergraduate curricula [9], one of the factors responsible for suboptimal antibiotic prescribing practices reported worldwide [10].

Efforts to support existing and future antibiotic prescribers have been evidenced by the burgeoning number of educational resources already developed [11]. However, most of these resources have focused on improving the technical knowledge of prescribers about infections or antimicrobials [12]. While such education would be undoubtedly useful and of some benefit, it may have overlooked increasingly recognized behavioral influences on antimicrobial decision-making [13]. Surveys of trainee doctors and students evaluating educational interventions, for example, have highlighted how professionals still felt hesitant about their competence in antibiotic prescribing [14,15]. Other studies have stressed the inaction reported by some clinicians to modify antimicrobial prescriptions [16], demonstrating the communication, negotiation, and emotional skills required for antimicrobial decision-making [17]. It would be unlikely for such skills to be nurtured by passive approaches based on providing knowledge, instead requiring active and dynamic educational experiences that would allow the performance of trainees and clinicians to be examined and reviewed [18].

The combination of increasing computing power, ubiquity of portable devices, and near-complete internet coverage affords an ever-growing reliance on interventions based on games as clinical training aids and simulations [19]. Software development has also benefited from an increased understanding of the behavioral determinants of clinical decision-making and heuristics [20], with existing software now able to address some of the challenges presented by the frequent lack of engagement with traditional quality improvement interventions [21,22].

**Figure 1:**
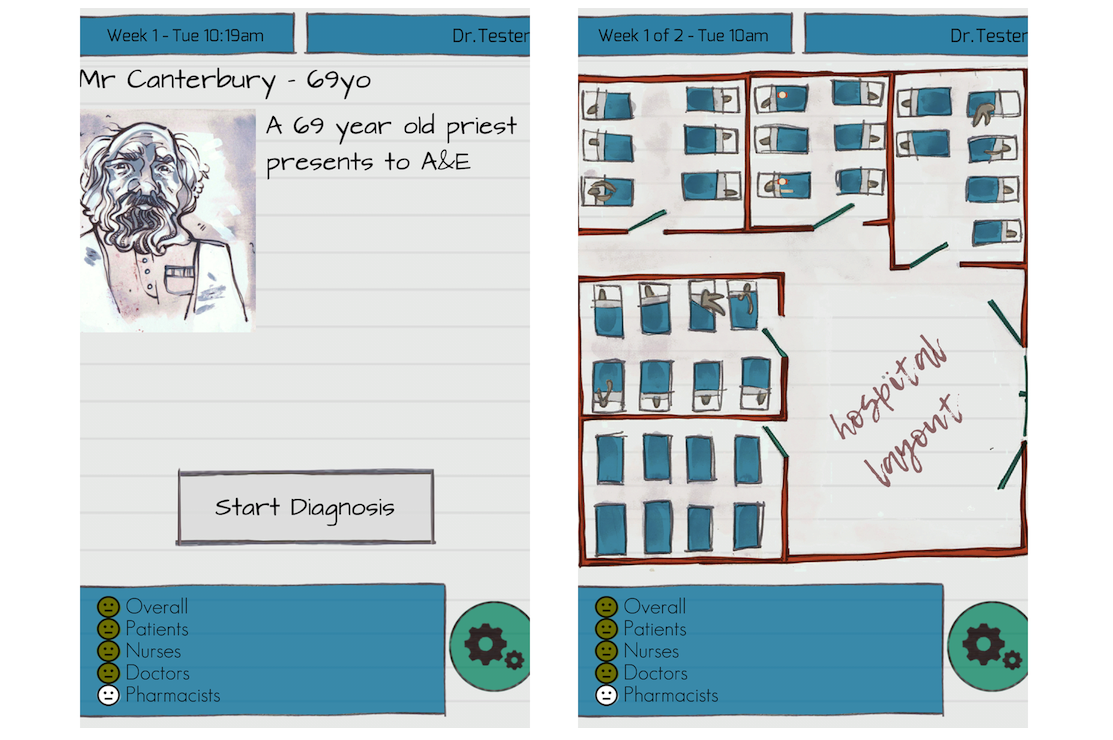
On call: Antibiotics. Interface (left panel); hospital layout (right panel).

Recognizing the potential of game-based solutions to facilitate education and training of health care students and existing clinicians involved in antibiotic management, in 2015 we developed On call: Antibiotics (OcA), the first serious game worldwide aimed at improving antimicrobial prescribing behaviors among hospital prescribers [23] (Figure 1 and Multimedia Appendix 1).

### On Call: Antibiotics Development and Use

A close collaborative of artists, commercial game developers, health care workers, academics, and patient representatives came together to design the game. The platform resembles clinical practice and presents a number of virtual patients that require management using essential diagnostic skills and the broad range of optimal behaviors embedded in established national antibiotic guidance in the United Kingdom such as Start Smart Then Focus (SSTF) [24], which offers these principles of optimal antibiotic use:

- Do not start antibiotics in the absence of clinical evidence of bacterial infection.
- For antibiotics prescribed, document each of the following on the drug chart and in the clinical notes: clinical indication (including disease severity if appropriate), dose, route, and duration or review date.
- Obtain cultures first where possible.
- Prescribe single-dose antibiotics for surgical prophylaxis where antibiotics have been shown to be effective.
- Review the clinical diagnosis and continuing need for antibiotics by 48 to 72 hours and make a clear plan of action: the antimicrobial prescribing decision.
- The five antimicrobial prescribing decision options are stop, switch, change, continue, and refer to outpatient parenteral antibiotic therapy.
- It is essential that the review and subsequent decision be clearly documented in the clinical notes. The decision should also be documented clearly on the drug chart.

Players are presented with demographic information about each patient (Figure 1, left panel) together with a chief complaint that can be explored by requesting more information about signs and symptoms. Once players are ready to make a diagnostic decision, they select a diagnosis and a therapeutic option according to the SSTF principles. As soon as the therapeutic decision is made, players are given feedback about their performance from the perspective of the different professional groups involved in antibiotic decisions. In addition to such individual feedback, metrics such as time engaged with the game and each case and time needed to make each decision are collected by the game and submitted to a secure, remote server for analysis.

The game does not aim to teach clinicians about specific antibiotics and their appropriateness or effectiveness to treat a given infection on the premise that such antibiotic-microorganism combinations are likely to change in time and be context-specific and that technical solutions (ie, those based on the provision of information or support to address a priori knowledge deficits), albeit effective, have been short-lived and demonstrated modest impact [25]. Instead, we were more interested in fostering excellent antimicrobial behaviors, recognizing that such optimal nature may depend on a variety of interrelated professional, clinical, and organizational conditions [26] and patient expectations [27].

To reflect the range of demands exerted on the different stakeholders within the antimicrobial prescribing pathway, we developed some archetypes or personae that embodied the goals of the different stakeholders. For example, the ideal outcome for patients may merely be the resolution of their infection in the fastest possible way, disregarding the potential impact that such a forceful approach may have on the rates of drug-resistant infections in the wider population. Hospital prescribers (in this version of the game, doctors), on the other hand, may strive for diagnostic and therapeutic accuracy, underpinning the wider reputation of the clinical team or the institution. Regarding the hospital, managers may have concerns about the use of costly intravenous medications and the increased number of patients developing adverse events associated with the peripheral vascular devices required to administer such antibiotics.

By definition, these archetypes are unidimensional and, to a point, simplistic, but they help articulate the tensions faced by prescribers. However, the foundation of such multifaceted perspective was the idea that satisfying all relevant stakeholders along the antimicrobial decision-making process may not be possible, and that even adequate therapeutic and clinical decisions may lead to negative consequences and experiences for some stakeholders. Additionally, using the personae ensured that although practitioners in the United Kingdom were the intended audience of the current game, health care workers worldwide involved in management of antibiotics in other countries could feel represented.

From game release on October 1, 2015, to September 2018, there were approximately 4000 downloads with about 2100 unique game users worldwide (source: website analytics, Google Data Studio reports). Our dissemination approach has been cautious until now, showcasing the game as a tool with the potential to influence antimicrobial prescribing among clinicians but avoiding any claims of efficacy or effectiveness until the results of pending evaluations are obtained.

Our previous literature review of serious games for infection prevention and control and AMS had identified a handful of reports within this field [28]. In their reporting, the majority of software and apps emphasized development aspects followed by the theoretical underpinnings of the products. However, few if any papers offered a detailed account of implementation, adoption, or evaluation perspectives and challenges. Such absence of published experiences seems to widely affect the field of serious games in medical and clinical education. For example, Gorbanev [29] recently explored the quality of the evidence of game effectiveness for medical learning, noting a weakness of diffusion mechanisms and lack of repeated implementations of games in different settings, highlighting how in general games were implemented, tested, and reported only once and in only one setting, with no comparator. Additionally, the unintended consequences of incorporating serious games as educational and behavioral tools in clinical education, including game elements and techniques as a source of distraction, have not been fully explored [30]. Addressing this gap in the evidence remains crucial to ensure meaningful impact in the real world, particularly important considering that these serious games intend to modify behaviors.

For such reasons, and to help identify potential threats to the successful deployment, implementation, and adoption of the game, we organized a co-development workshop with clinicians and academics in serious games and antimicrobial use to debate those gaps and co-design solutions.

## Methods

### Attendee Selection

The workshop was widely advertised on our institutional website, event-booking platforms, and social media. We reviewed all individuals registered to attend the event and rejected people who could not demonstrate clinical expertise in antibiotic use or experience developing serious games or researching the theoretical and behavioral frameworks included in serious games to justify their attendance. We added such evaluation to ensure that room capacity for the event was not surpassed. Ideal participants were involved in prescribing, reviewing, or administering antibiotics; developing serious and other types of games; or developing or researching behavioral methods or tools, regardless of whether they focused on digital or nondigital delivery approaches.

### Purpose of the Workshop

To maximize participation and cost efficiency, the workshop took place prior to the International Summit on Serious Health Games [31] organized by the Health Protection Research Centre for Healthcare Associated Infection and Antimicrobial Resistance at Imperial College London on March 20, 2018, to showcase innovative serious health games that had demonstrated robust or imaginative methodologies during their development, implementation, or evaluation or focused on topics transferable to the purpose of our game.

The 2-hour workshop was arranged from a perspective of co-design and coproduction, although we recognized that participants would be presented with a fairly stable and defined version of the game, allowing no modification of either the user interface or functionality. The layout of the workshop venue was arranged so attendees could work in small groups around a laptop or tablet running the game following brief presentations from the workshop organizer (ECS). The organizer summarized responses from each small group, seeking their agreement about the fidelity of such summary and noting divergent opinions on flipcharts. Workshop participants could also download the latest game version on their personal devices from the institutional repository [32].

We urged attendees to hypothesize whether the use of the game could lead to unintended consequences or unexpected events. Reflecting the gaps that had been identified in the literature review, experts were asked to sequentially debate the challenges in implementation, adoption, and evaluation of OcA, as highlighted in the manuscript by Castro-Sanchez et al [28]. For example, among the adoption challenges, participants had to identify the strengths, weaknesses, opportunities, and threats for clinicians, end users, technologists, developers, and those responsible for funding or commissioning the game. Regarding implementations challenges, participants were specifically asked to reflect upon the scalability and sustainability of OcA as a learning and behavior modification tool for clinicians. Finally, the section on evaluation challenges requested that workshop participants identify qualitative, quantitative, mixed, and economic evaluation approaches from the point of view of clinician players and technologists or developers engaged in the evaluation, with ideas about the type of evidence funders and commissioners may wish to receive to approve implementation and adoption of the game across new organizations.

Participants were encouraged to consider perspectives ranging from the individual clinician to the organization and the health service. They were asked to consider potential challenges to translating the intervention to low-resource settings to allow it to run on virtually any computing platform and focus its design away from particular therapeutic regimens and toward behaviors that have been agreed upon in many settings already. Although the workshop preceded the Summit, attendance was voluntary.

### Analysis

As the workshop had a coproduction perspective, the goal was to identify actionable solutions that could be iterated in real-world use of the game and refined rather than establishing hypotheses or exploring perceptions or ideas of the participants about the implementation of the game. Threats and solutions were summarized by the workshop leader from opinions expressed during the workshop and by each team of participants after each of the workshop sections. These summaries were corroborated or further clarified by the participants and collectively agreed. Notes from the observer also documented interactions between participants and emerging interesting ideas. This paper synthesizes threats and solutions generated during the workshop.

## Results

### Characteristics of Participants

Of the applicants, 29 were selected to attend the workshop, including consultant physicians from different clinical specialties; doctoral and postdoctoral researchers with projects focused on simulation, games, or virtual environments; antimicrobial resistance researchers and clinicians; experts in digital intervention implementation; games developers; behavioral researchers with interests in game-based interventions; and funding and digital project managers. Workshop members came from the United Kingdom, Spain, Germany, Portugal, France, and Ireland. Table 1 summarizes the solutions coproduced at the event.

**Table 1:** Solutions proposed by workshop attendees.

Stage	Threat	Solution
Implementation	Inadequate organizational set-up for scalability and sustainability (technical and educational support for players and others, data handling, workforce volatility)	Consult practice educators and tutors on future game versions to preserve professional and game pedagogies aligned
Adoption	Perceptions about games as ludic rather than serious tools; questioning professionalism of game users	Identification of optimal game users and timing. Embedding game in other training and education solutions
Evaluation	Unsatisfactory evaluation frameworks so added value of games to existing multimodal bundles cannot be robustly measured	Multiple mixed-methods evaluations (quantitative, qualitative, and in-game) that provide added value to existing tools; identification and linkage to in-practice and cost-relevant metrics

### Implementation Challenges

The most pressing implementation challenges identified by participants referred to the organizational setup necessary to ensure scalability and sustainability of the game as a viable professional tool. For participants, successful implementation of OcA would require the release of the game underpinned by robust technical and educational support for players, with inclusion of other nonplaying clinicians and any other professionals in education in practice.

Ideally, these educationalists or practice educators and tutors would remain consulted about any future game iterations so required learning or professional outcomes are reflected in the game. Similarly, education and training leads would receive intelligence from in-game metrics and a variety of proposed evaluation results to maintain a coherent and aligned pedagogy.

The scalability of the game as a platform was not really considered to be a threat to the success of the intervention, as the total potential number of gamers at the institution would not be overwhelming to the resources in place to host the software. Instead, attendees felt that, potentially, the volume of data generated by the game itself combined with clinical information and experiential gamer feedback may end up being overwhelming for organizational follow-up and lack sufficient granularity to enable continued practice improvement. As an example, attendees wondered how it would be possible to maintain personalized feedback, training, and education for each of the players, potentially most medical prescribers in the hospital.

Linked to the previous concern but in terms of sustainability, the cyclical nature of the UK trainee medical workforce targeted, transferring from one health care organization to another every few months, may obfuscate the meaningful follow-up of players. What should be done, for example, when clinicians leave the organization yet remain engaged with the software to avoid their in-game generated data confuse decisions by the hospital management?

### Adoption Challenges

Attendees agreed that adoption of a game-based intervention such as OcA as an adjuvant to support clinical practice may suffer from perceptions about serious games as valid behavioral tools. As suggested previously, potential end users may reject the tool simply on the basis of their ideas about a game as a ludic experience and not the proper training required to address the assumed lack of knowledge about antibiotic guidelines, considered to be the ultimate reason for inappropriate prescribing.

Participants added that to encourage game adoption it would be necessary to make explicit who should be using the game and the timing of its use. Such unambiguous guidance would also be welcomed as OcA, albeit reflecting clinical realities, was not a simulator (ie, a life-like representation), like other tools such as surgical training platforms, or readily available, like advanced life support stations.

Regarding the characteristics of the ideal candidate players, clinicians unfamiliar with the demographics of gaming may assume that games would just be appealing or valid for, generally speaking, male and younger health care professionals. These perceptions, however, may not be aligned with existing evidence. Regarding the timing of game use, the workshop attendees suggested that potential end players may hesitate to be seen engaging with the game during working hours for fears of doubts about their professionalism. Such perceptions should not be dismissed and could be robust enough to derail the sustained adoption of OcA and similar tools.

**Figure 2:**
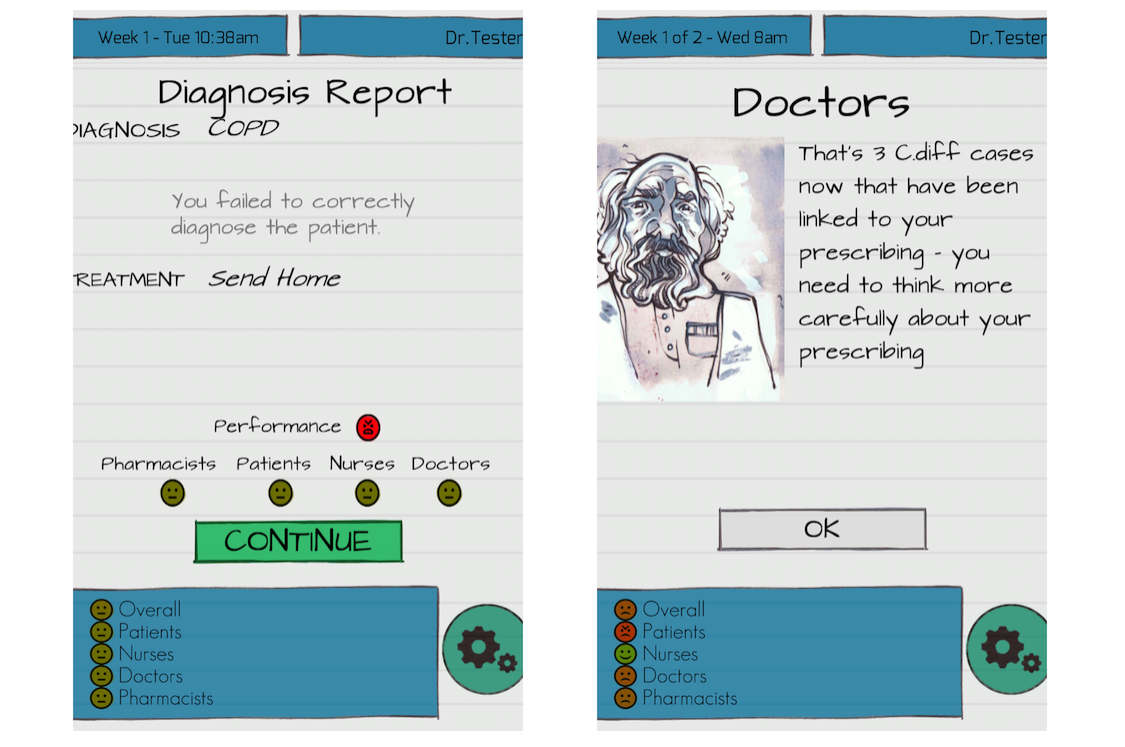
In-game feedback. Immediate performance (left panel); cumulative performance (right panel).

To mitigate such threats, attendees advocated for an adequate communication campaign about the evidence underpinning the use of the game and the inclusion of the game within the pool of training and continuous professional development interventions offered by the organization. For instance, there should be concerted efforts to integrate this game-based platform among established performance review mechanisms such as portfolios, embedding game scores and feedback or using in-game performance as reflection to be jointly discussed between assessors and appraisees to evaluate skills and decision-making (Figure 2).

Interestingly, some attendees highlighted the dilemmas that may arise when proposing the use of games as interventions aimed at resolving attrition and lack of engagement. Although unlikely, it was hypothesized that extremely successful initiatives may nudge end users to play too much or at inappropriate times.

### Evaluation Challenges

Attendees agreed on the clear need to ensure that game-based behavioral tools are robustly evaluated, ideally using different metrics and approaches, if they are to be postulated as effective and worthy of adoption by clinicians and funding by commissioners and decision-makers. Ideally, evaluations should be based on the most stringent yet feasible research design and focused on matching the game against current educational interventions or in addition to such interventions so the added value to existing multimodal bundles can be measured.

In addition to outcome-oriented evaluations, participants stressed the benefit of qualitative or mixed-methods designs that facilitated discussions with potential players about their embedding of serious games within professional or personal workflows, synergies with existing education or training approaches, and sustainability of perceived behavior changes.

**Figure 3:**
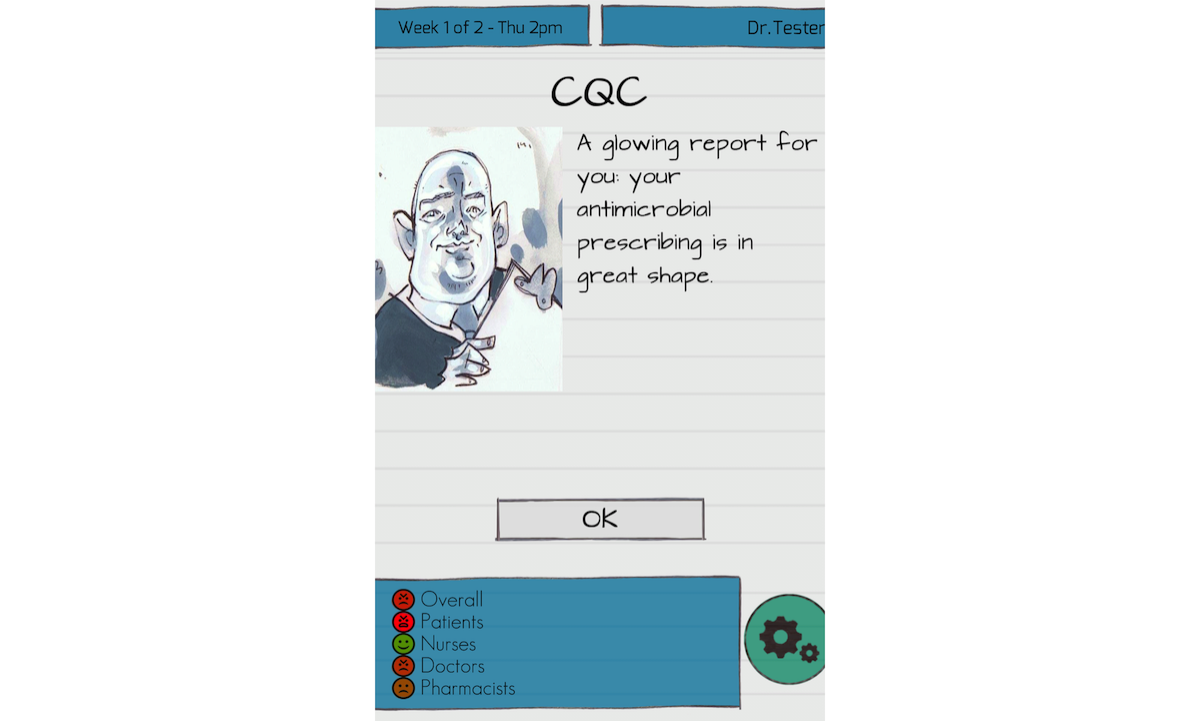
In-game behavioral nudge.

For attendees, evaluation design depended on the niche identified for the game. As the game aspired to improve suboptimal prescribing behaviors, it would therefore be crucial to consider with care which outcome evaluation indicators to appoint. Further, as OcA introduced multiple perspectives regarding the impact of prescribers’ decisions, agreeing on what optimal prescribing represented may be complex (Figure 3). Of interest, planned OcA evaluation activities include randomized controlled trials complemented by semistructured interviews triangulated with in-game metrics.

Two final notes of caution were mentioned during the workshop. First, there were concerns about how to best link behavioral and clinical metrics regarding outcome evaluation. As in-game performance had to be matched to clinical, real-world performance, there would be a need to establish how individual behaviors in the game would be linked to performance reflected on clinical records. Obviously, this challenge would only be relevant if the unit of analysis for the adopted evaluation design focused on individual clinicians; should other designs center on wards or teams, other metrics may be preferable or suitable. Second, there were concerns about how to determine any cost-related benefits in view of the complexity to arrive confidently at game-attributable improvements in clinical practice.

## Discussion

### Principal Findings

The potential of innovative game-based interventions in clinical practice can be threatened by perceptions about the validity of such behavior change approaches, as reported by the attendees to our workshop. To mitigate such threat, game advocates ought to include explicit guidance and information in their implementation strategies about intended end players and when they could use the game. Ideally, games should be positioned as equal tools among other interventions, with efforts to build synergies between tools and approaches and, ultimately, work toward a multichanneled ecosystem of educational experiences.

To achieve such parity with traditional resources, our attendees recommended that games should undergo robust evaluations from multiple perspectives with the aspiration to achieve noninferiority against already established resources. Evaluations should focus on clinical rather than statistical success. The emphasis on real-world improvement would facilitate the assessment of cost-associated measures in order to convince decision-makers of the benefits of deploying the game.

### Limitations

The main limitations affecting the results refer to the very circumscribed software discussed at the workshop, a serious game focused on improving clinical behaviors among prescribers in the United Kingdom; the moderate number of attendees to the workshop; and the format of the event, where opinions where sought and had to be offered openly following a brief period of contact with the game, which may have contributed to socially desirable responses. Other academics or software coders interested in developing game-based solutions to influence clinical behaviors may still benefit from our findings, particularly the prominence given to adequate evaluation designs, including qualitative approaches.

### Comparison With Prior Work

Beside OcA, few if any serious game-based approaches have been published on antimicrobial stewardship. However, our workshop centered on mitigating any expected or foreseen implementation, adoption, and evaluation challenges likely to arise when deploying a game-based intervention in clinical practice.

From that perspective, our results complement work about technology implementation [33] by suggesting that organizations interested in games would have to dispel concerns about sensitivities on their appropriateness as valid educational instruments, affecting the brand attitude [34] and modulating the intention to use factor predicated within models such as the technology acceptance model [35] or technology integration model [36].

Further, although perceived ease of use has been recognized as a contributing factor toward games adoption, our results suggest that it would be paramount to consider such ease not just from the game itself but also from the embedment of the game within the existing continuous professional development and educational workflows gamers may have already in place. As OcA can link to professional portfolios and output gaming hours and performance scores easily, the integration into the workflow may not be that difficult, which may not be the case for every game and would require careful planning [37].

Finally, our attendees firmly endorsed robust and multiple evaluation approaches, a concern well described previously [38]*.* However, they stressed the economic aspects necessary to convince decision-makers about the use of the game, perhaps reflecting wider difficulties of introducing novel technologies onto clinical practice and education at times of funding restrictions.

### Conclusions

Game-based interventions can aid efforts to improve antimicrobial use, but their successful deployment and sustained use cannot rely on assumptions about any inherent interest to clinicians. Even if accurate, such assumptions would need to resolve perceptions about the use of games as professional learning platforms and be supported by organizational planning and multipronged metrics of success.

## Appendix

Multimedia Appendix 1 “On call: Antibiotics”, a first antimicrobial stewardship behaviour change serious game.

## Abbreviations

AMS: antimicrobial stewardship
NIHR: National Institute for Health Research
OcA: On call: Antibiotics
SSTF: Start Smart Then Focus
